# Cementing CO_2_ into C-S-H: A step toward concrete carbon neutrality

**DOI:** 10.1093/pnasnexus/pgad052

**Published:** 2023-03-28

**Authors:** Damian Stefaniuk, Marcin Hajduczek, James C Weaver, Franz J Ulm, Admir Masic

**Affiliations:** Department of Civil and Environmental Engineering, Massachusetts Institute of Technology, 77 Massachusetts Ave, Cambridge, MA 02139, USA; Department of Civil and Environmental Engineering, Massachusetts Institute of Technology, 77 Massachusetts Ave, Cambridge, MA 02139, USA; Wyss Institute for Biologically Inspired Engineering, Harvard University, 3 Blackfan St, Boston, MA 02115, USA; Department of Civil and Environmental Engineering, Massachusetts Institute of Technology, 77 Massachusetts Ave, Cambridge, MA 02139, USA; Department of Civil and Environmental Engineering, Massachusetts Institute of Technology, 77 Massachusetts Ave, Cambridge, MA 02139, USA

**Keywords:** cement, concrete, carbonation, chemomechanics, Raman spectroscopy, indentation

## Abstract

Addressing the existing gap between currently available mitigation strategies for greenhouse gas emissions associated with ordinary Portland cement production and the 2050 carbon neutrality goal represents a significant challenge. In order to bridge this gap, one potential option is the direct gaseous sequestration and storage of anthropogenic CO_2_ in concrete through forced carbonate mineralization in both the cementing minerals and their aggregates. To better clarify the potential strategic benefits of these processes, here, we apply an integrated correlative time- and space-resolved Raman microscopy and indentation approach to investigate the underlying mechanisms and chemomechanics of cement carbonation over time scales ranging from the first few hours to several days using bicarbonate-substituted alite as a model system. In these reactions, the carbonation of transient disordered calcium hydroxide particles at the hydration site leads to the formation of a series of calcium carbonate polymorphs including disordered calcium carbonate, ikaite, vaterite, and calcite, which serve as nucleation sites for the formation of a calcium carbonate/calcium-silicate-hydrate (C-S-H) composite, and the subsequent acceleration of the curing process. The results from these studies reveal that in contrast to late-stage cement carbonation processes, these early stage (precure) out-of-equilibrium carbonation reactions do not compromise the material's structural integrity, while allowing significant quantities of CO_2_ (up to 15 w%) to be incorporated into the cementing matrix. The out-of-equilibrium carbonation of hydrating clinker thus provides an avenue for reducing the environmental footprint of cementitious materials via the uptake and long-term storage of anthropogenic CO_2_.

## Introduction

Concrete, with its main component, ordinary Portland cement (OPC), is the most widely used construction material in the world. First introduced in the early 19th century, OPC is a mixture of limestone and clay, which is calcined at high temperatures (∼1,450°C) and ground to produce a multiphase clinker, consisting of alite (*C*_3_*S*, (*CaO*)_3_*SiO*_2_) and belite (*C*_2_*S*, (*CaO*)_2_*SiO*_2_). Due to a combination of factors including its widespread use, the extreme temperatures required to stabilize clinker phases, and the carbon-positive process of limestone (CaCO_3_) calcination to produce CaO, OPC production currently accounts for ca. 8% of global CO_2_ emissions (1). Most countries worldwide have defined roadmaps to achieve carbon neutrality by 2050, and in the case of cement-associated emissions, one major focus has been to create concrete that can function as a carbon sink (2, 3). Various initiatives have investigated the potential for mineralizing CO_2_ into concrete via a process known as carbonation (4–6). Combining these efforts with the utilization of other alkaline mineral waste products from the metal refining, fuel combustion, and manufacturing industries has the potential to significantly impact global carbon sequestration efforts (7). Because of the intrinsic alkaline nature of the products formed from clinker hydration, hardened cement already serves as a significant atmospheric CO_2_-absorbing agent through the natural carbonation of calcium hydroxide (portlandite, CH). In fact, recent modeling results have revealed that, through this process of late-stage carbonation, the cumulative uptake of CO_2_ can, over time, be equivalent to ∼43% of the limestone-related OPC production emissions (8).

In an alternate approach, forced carbonation, which occurs via the localized increase in the availability of CO_2_ during the early stages of cement hydration, offers great potential as an additional CO_2_ sequestration mechanism (9–11). It should be noted, however, that these early stage carbonation reactions are fundamentally different from those that occur during late-stage carbonation, where the destabilizing nature of cured OPC carbonation leads, through a series of phase transformations, to a drop of the pore solution pH, material shrinkage, and a reduction in structural integrity via the formation of microcracks (4, 5, 12, 13). The process of cement forced carbonation ultimately leads to the formation of gel-like C-S-H phases, which are generally intermixed with various metastable polymorphs of calcium carbonate (disordered CaCO_3_ (DCC), vaterite, and aragonite). If in excess, dissolved CO_2_ can further react with C-S-H, leading to the formation of a mixture of CaCO_3_ and silica gel (4, 14). The forced carbonation-induced accelerated curing of fresh concrete, in contrast to natural carbonation of mature concrete, shows significant benefits in terms of improved strength, reduced water absorption, improved resistance to chloride permeability, and improved freeze–thaw performance (15).

Through the development of new high-throughput correlative imaging and chemical mapping tools, it is now possible to explore these processes over much shorter time scales, revealing previously unknown intermediate phases and maturation pathways (16–18). For example, a recent time-resolved investigation of the early stages (first 24 h) of cement hydration in the absence of induced carbonation (16) identified the presence of a transient disordered CH phase (DCH) that forms before setting begins (during the precure stage), which over time matures into crystalline pore-filling portlandite. These transient, mostly overlooked phases might therefore offer opportunities to explore out-of-equilibrium processes associated with precure stage carbonation in freshly formulated cements, and as such, could be leveraged to sequester large quantities of CO_2_ in the form of stable carbonate minerals. While the potential of forced carbonation reactions to be employed for CO_2_ sequestration in a postcuring/postsetting materials context (forced carbonation and late-stage natural carbonation) has been well documented (5–7, 11, 12, 14, 19, 20), very little is known regarding the time-resolved multiscale evolution and out-of-equilibrium transformation of specific mineral phases that occur during precuring/presetting stage carbonation or the mechanical consequences of these reactions. In order to better understand the underlying multistep mechanisms of precure carbonation reactions, we applied a correlative time- and space-resolved Raman microscopy and indentation approach to study the chemomechanics of cement carbonation over time scales ranging from the first few hours to several days. We show that precure carbonation of cement is not associated with the deterioration of the material's structural integrity and leads to a significant incorporation of CO_2_ (up to 15 w%, as estimated from TGA) in the form of calcite and a CaCO_3_/C-S-H composite phase. At the nanoscale, this composite phase consists of C-S-H, calcite, and disordered calcium carbonate (DCC), which are formed through the carbonation of transient DCH particles at the hydration site. At the micron level, the composite phase incorporates a series of calcium carbonate polymorphs (including ikaite and vaterite) that eventually convert into calcite, the most stable form of CaCO_3_.

## Results and discussion

### Early stages of the precure carbonation process

**Fig. 1.**
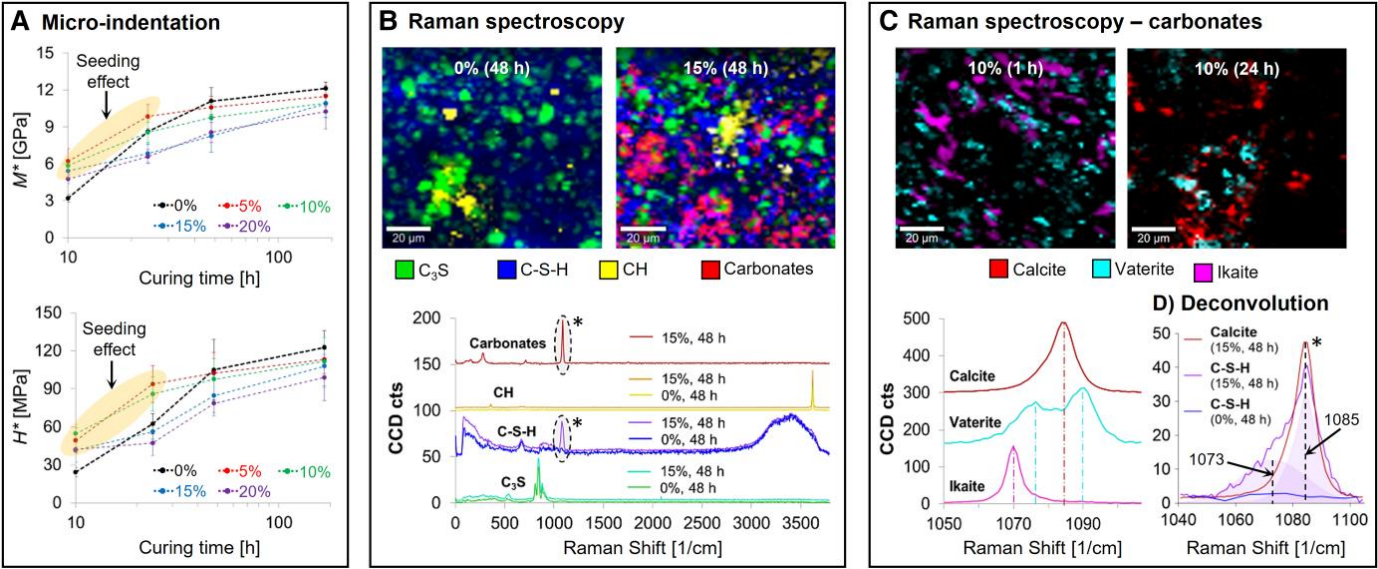
Chemomechanics of early stage C_3_S carbonation. A) Time evolution of the specific indentation modulus (M*) and hardness (H*) in bicarbonate-substituted cements. B) Phase maps of alite and its hydration products and their corresponding Raman spectra (sample 0 and 15% at 48 h of hydration). C) The formation of calcite, vaterite, and ikaite at different stages of hydration (sample 10%, 1 and 24 h) and their characteristic *v*_1_ vibrational modes. D) Deconvolution (from the regions denoted by asterisks in (B)) of the carbonates group in C-S-H/carbonates composite (with 15% bicarbonate substitution, 48 h), showing the coexistence of calcite and DCC.

In order to elucidate the underlying chemomechanical changes that occur during precure carbonation processes in cementitious materials, we developed a model system that consisted of sodium bicarbonate-substituted cement formulations to mimic the conditions that exist following CO_2_ enrichment. The micro-indentation-based time evolution of the specific indentation modulus (M*) and hardness (H*) of bicarbonate-substituted cements is reported in Fig. 1A. During the early stages of hydration (first 10 hours), all bicarbonate-substituted samples exhibit significantly higher M* and H* compared with the bicarbonate-free control (with a 2-fold increase in mechanical performance in the case of sample with 5% of bicarbonate substitution), evidencing a clear nucleating effect of the carbonates (which are derived via bicarbonate deprotonation) and subsequent strengthening of the hydrating clinker. While after 7 days, the control sample slightly mechanically outperforms its carbonated equivalents, no statistically significant difference in terms of hardness can be observed for samples with up to 15% bicarbonate substitution. Statistical analysis was performed using the *T*-test at a significance level of 5%.

### Effects of bicarbonate substitution

The time- and space-resolved confocal Raman microscopy (CRM) studies of the early stages of hydration (Fig. 1B) allowed the tracking of carbonate-related phase transformations in the bicarbonate-substituted samples. In addition to C_3_S, C-S-H, and CH formation, which are typical for the negative (bicarbonate-free) control, the presence of a series of calcium carbonate polymorphs and calcium carbonate hydrates can be seen. For example, in the 10% bicarbonate-substituted sample (Fig. 1C), calcite, vaterite, and ikaite (CaCO_3_·6H_2_O) can be clearly distinguished on the basis of the *v*_1_ carbonate vibrational mode (21). Previously unreported ikaite was observed during the earliest stages of hydration (in the first 5 h) for all bicarbonate-substituted samples, whereas calcite and vaterite dominated the carbonate-related species after 5 h of hydration, and only calcite was observed after 7 days. Furthermore, CRM revealed the presence of a CaCO_3_/C-S-H composite phase (Fig. 1B starred), where the coexistence of calcite and DCC (a broad shoulder in the 15%, 48 h spectrum in Fig. 1D) suggests their potential role in the nucleation of C-S-H and the co-precipitation of the CaCO_3_/C-S-H composite.

### Effects of forced carbonation on C-S-H formation

**Fig. 2.**
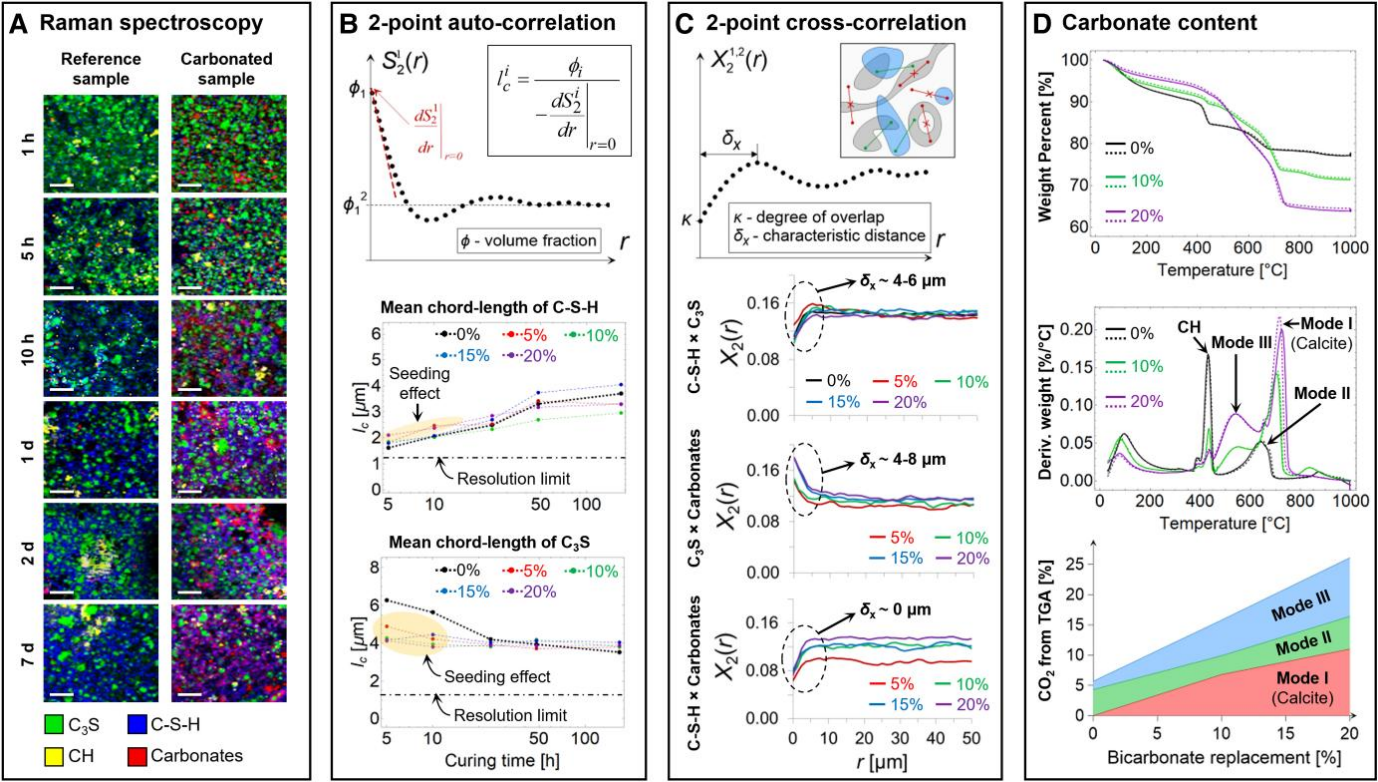
Time- and space-resolved chemomechanics of C_3_S hydration and carbonation. A) Temporal evolution and detailed relationships between different mineral phases formed for samples with and without bicarbonate substitution. Scale bars represent 20 µm. B) The mean chord-length (*l*_c_) calculated on the basis of the volume fraction and the slope at the origin (*r* = 0) of the two-point auto-correlation function (*S*_2_(*r*)) for C-S-H and clinker phases. C) Two-point cross-correlation function (*X*_2_(*r*)) at 48 h of hydration between C-S-H and C_3_S, C_3_S and carbonates, and C-S-H and carbonates. (D) CO_2_ weight loss for different bicarbonate substitutions at 3 months of hydration estimated on the basis of thermogravimetric analysis.

In order to investigate the temporal evolution and detailed spatial distribution of the different mineral phases formed during early stage carbonation (Fig. 2A), we applied correlation function analysis on the CRM phase maps (Fig. 2B-C). The chord length (*l*_c_), which reflects the average particle size (Fig. 2B), is appreciably higher for C-S-H in the bicarbonate-substituted samples at the early stages of hydration, compared with the controls. This increase in hydration kinetics is reflected in a strength gain in the bicarbonate-substituted samples (Fig. 1A) due to a nucleating/seeding effect. The 2-point cross-correlation functions evaluated at 48 h of hydration (Fig. 2C) show that the characteristic distance (*δ*_x_) between C-S-H and clinker, and clinker and carbonates is in the range of 4–6 and 4–8 μm, respectively, while for C-S-H and carbonates, *δ*_x_ = 0. These results clearly demonstrate the spatial co-precipitation of C-S-H and carbonates and a comparable spatial relationship between clinker and both C-S-H and carbonates phases. Consequently, the degree of overlap (*κ*) is the highest for C-S-H and carbonates (*κ* in the range 0.14–0.18), when compared with C-S-H and clinker (*κ* ∼ 0.12), or clinker and carbonates (*κ* in the range 0.06–0.08). Furthermore, the extent of bicarbonate substitution scales with the mass fraction of CO_2_ related weight loss estimated from TGA analysis (Fig. 2D) and therefore provides further insights into the identity of the carbonation products. For example, in addition to the 5% of carbonates associated with the C_3_S reference sample (mode II in Fig. 2D), we observe two main carbonate polymorphs, namely calcite (mode I in Fig. 2D) and amorphous calcium carbonate (mode III in Fig. 2D), which both scale equally with the extent of bicarbonate substitution. If we assume that the amount of mineralizable CO_2_ is strictly related to the amount of DCH available for carbonation, we are able to estimate the theoretical CO_2_ uptake capacity from the forced carbonation process. If the hydration of clinker leads to a high Ca/Si ratio C-S-H (Ca/Si = 1.7), then the theoretical offset of CO_2_ amounts to at least 40% of carbon emissions from cement production, not including emissions associated with fossil fuels used in the process (for more details, see Material and methods section).

## Discussion

**Fig. 3.**
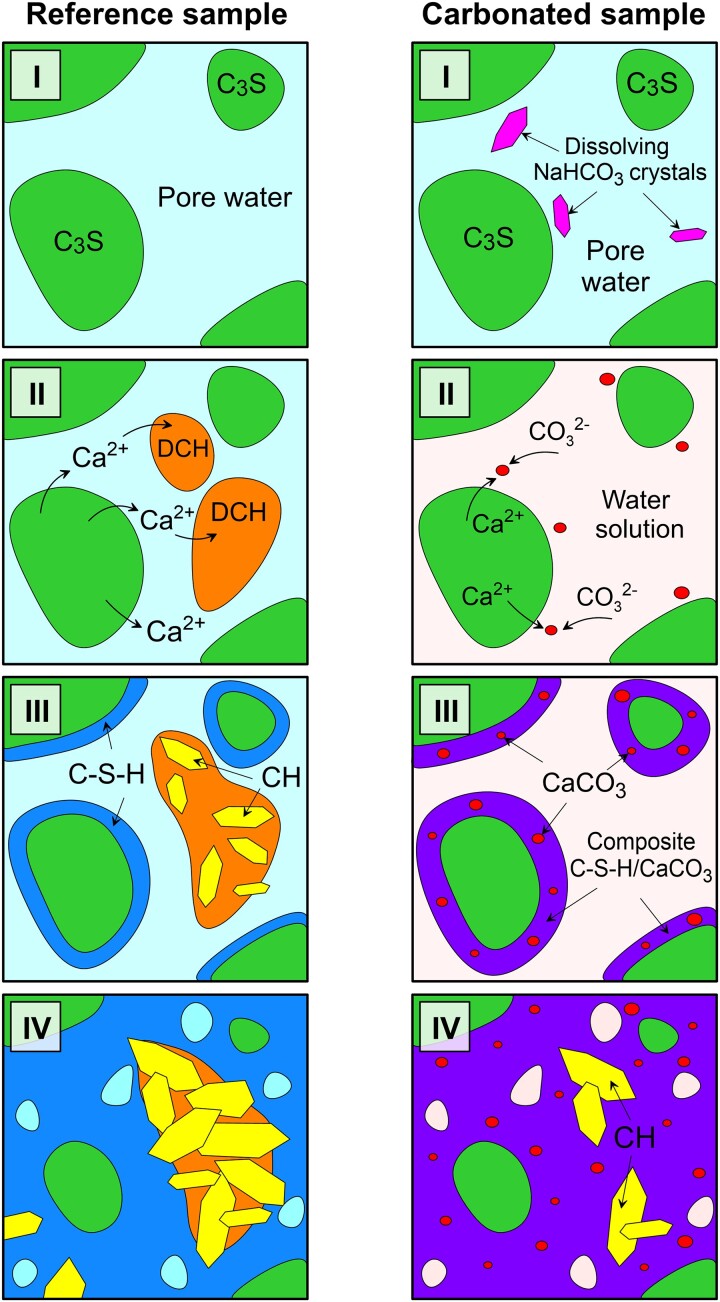
A schematic representation of early stage hydration reactions (precure I-III and postcure IV) for samples with and without bicarbonate substitution (the colors correspond to the Raman map data in Fig. 2A).

By combining these results, we can begin to explain the processes of early stage (pre- and postcure) clinker carbonation (Fig. 3). In the bicarbonate-free controls, C_3_S slowly hydrates to produce C-S-H and DCH, and nanoparticles of DCH migrate into the pore spaces and crystallize. In the bicarbonate-substituted samples, the presence of carbonate ions at the hydration sites (the regions of direct water-clinker contact) leads to an out-of-equilibrium conversion of the amorphous DCH particles into amorphous DCC, which in turn, serves as a nucleation site for the formation of C-S-H, leading to a series of subsequent changes: (i) the formation of a CaCO_3_/C-S-H composite phase; (ii) the acceleration of clinker hydration, and its subsequent (iii) increased early stage mechanical performance. Since this out-of-equilibrium carbonation takes place in the freshly mixed stage before solid percolation, the mineralized carbonates effectively serve as sites for permanent CO_2_ storage in the CaCO_3_/C-S-H composite, allowing a partial offset (up to 15%) through mineralization of the carbon emissions associated with clinker production and leading to a material with improved early stage mechanical performance.

While the long-term durability of these out-of-equilibrium composites has yet to be fully characterized, the results from our mechanical testing studies suggest that the introduction of precure stage carbonation could simultaneously be harnessed as a critical carbon sink, while potentially mitigating some of the detrimental mechanical consequences of late-stage carbonation by shifting these reactions to an earlier time point where internal stresses can be relieved before they can destructively accumulate. The implementation of multiscale and time-resolved chemomechanical studies such as those reported here can thus provide critical insights into the maturation pathways of cementitious materials, while identifying new chemistries that can be effectively leveraged for combining CO_2_ sequestration with longer-term material durability in the built environment.

## Materials and methods

For all samples, pure alite (C_3_S) was combined with different sodium bicarbonate (NaHCO_3_) substitutions [0 (control sample), 5, 10, 15, and 20%]. For mechanical analysis, instrumented micro-indentation was employed, and Raman spectra at different curing ages (1 h, 5 h, 10 h, 1 day, 2 days, and 7 days) were acquired from a CRM system (Alpha 300RA; WITec, Germany). The chemical maps obtained by CRM were analyzed using correlation functions to quantitatively represent the phase distributions and their spatial correlation at progressing stages of hydration. The theoretical capacity of the forced carbonation process was determined on the basis of a stoichiometric analysis. The extended materials and methods section can be found in the supplementary information.

## Supplementary Material

pgad052_Supplementary_Data

## Data Availability

All relevant data are included in the article or supplementary material.
