# Supplementary material for: Cementing CO2 into C-S-H: A step toward concrete carbon neutrality
Source: PNAS Nexus. 2023 Mar 28;2(3):pgad052. doi: 10.1093/pnasnexus/pgad052 (PMC10062303; doi:10.1093/pnasnexus/pgad052)
Supplement: pgad052_Supplementary_Data [file pgad052_supplementary_data.pdf]

Supplementary Information for

Cementing CO_2 into C-S-H: A step toward concrete carbon neutrality

Damian Stefaniuk[1], Marcin Hajduczek[1], James C. Weaver[2], Franz J. Ulm[1], Admir Masic[1,*]

[1]Department of Civil and Environmental Engineering, Massachusetts Institute of Technology, 77 Massachusetts Ave, Cambridge, MA 02139, USA

[2]Wyss Institute for Biologically Inspired Engineering, Harvard University, 3 Blackfan St, Boston, MA 02115, USA

*Admir Masic
Email: masic@mit.edu

This PDF file includes:

Supplementary text
Caption for Dataset S1
SI References

Other supplementary materials for this manuscript include the following:

Dataset S1

Supplementary Information Text

Extended methods

Materials. For all samples, pure alite (C_3S) (chosen for its much simpler chemistry compared to the more heterogeneous OPC) was obtained from Mineral Research Processing (Meyzieu, France), and combined with five different concentrations of sodium bicarbonate ($NaHCO_3$): 0% (control sample), 5%, 10%, 15%, and 20%. A water:solid (w/s) ratio of 0.42 was used for the 0%, 5%, and 10% $NaHCO_3$ samples, and 0.43 and 0.44 w/s for the 15% and 20% samples, respectively, to maintain comparable sample workability.

Micro-indentation. Before testing, all samples were first cut to size with a water-lubricated slow-speed diamond saw (which washed away any residual Na), and wet surface-polished using sequential silicon carbide grinding papers with P2500 and P4000 grits, and dry aluminum oxide abrasive discs with sequential particle sizes of 3 μm and 1 μm. For mechanical characterization of the different samples, instrumented micro-indentation (Anton Paar Instruments) was employed. All measurements were carried out with a Berkovich tip, and anomalous force–displacement curves resulting from indentations at locations containing surface cracks were removed from the analysis. For all data sets, indentation hardness H and indentation modulus M were calculated using the Oliver-Pharr method (1). All specimens were loaded in force-control up to the limiting maximum indentation depth of 24 μm at a loading rate of 3 N/min. At peak load, the force was held constant for 10 s before the load was removed at a rate of 3 N/min, which was fast enough to avoid creep effects that can affect the elastic unloading. 15 to 30 microindentation measurements were performed at each curing age (10 h, 1 day, 2 days, and 7 days) to analyze the change in mechanical properties due to an addition of $NaHCO_3$. The microindentation results are presented in terms of specific indentation modulus ($M^*=M/c$) and specific indentation hardness ($H^*=H/c$), where c is the mass of alite divided by the total mass of solids (e.g., c=1 for the reference samples, and c=0.8 for the samples with 20% replacement of $NaHCO_3$).

Raman spectroscopy. A Confocal Raman Microscopy (CRM) system (Alpha 300RA; WITec, Germany) was used to obtain Raman spectra at different curing ages (1 h, 5 h, 10 h, 1 day, 2 days, and 7 days) using a Nd:YAG laser (λ = 532 nm) and a 63× Zeiss water immersion objective for underwater measurements. The excitation wavelength was calibrated using a silicon wafer standard. Raman maps of 100×100 μm scan areas (80×80 points) were acquired with a continuous laser beam and an accumulation time of 0.2-0.3 s per point. At least three maps at each hydration step were acquired in order to ensure the representativeness of the results.

For the Raman spectroscopy measurements acquired at time points up to 24 h, no sample preparation was required. After mixing, each sample (while still in a slurry-like state) was placed on a glass microscope slide and the top surface was covered with a quartz coverslip to prevent the sample from drying and to eliminate its contact with the surrounding environment. After 24 h (after which the samples had solidified), sample preparation followed the same procedure used for micro-indentation measurements, as described above.

The chemical maps obtained by CRM were analyzed using correlation functions (two-point auto-correlation ($S_2(r)$) and cross-correlation ($X_2(r)$) functions) to quantitatively represent the phase distributions and their spatial correlation at progressing stages of hydration. WITec Project and Matlab software were used for data analyses and visualization. More details regarding the applied methodology can be found in (2).

Thermogravimetric analysis. To assess the quantity of precipitated carbonates, thermogravimetric analysis (TGA) was employed. After 3 months of curing, the reference sample and those with 10% and 20% of bicarbonate substitution were ground to a fine powder, and then heated in air at a uniform rate of 5℃/min from 25 to 1000℃. For each sample, two replicates were performed using 25-30 mg of material. Three modes of CO_2 dissociation were distinguished following the work of Thiery *et al*. (3). Mode I, characterized by a peak in the range of 700-750℃, corresponds to well-crystallized carbonate, i.e., calcite. Modes II (600-700℃) and III (450-600℃) are associated with amorphous and less crystalline carbonates. The quantity of CO_2 (by mass) for each mode was calculated using deconvolution (based on Gaussian distributions) of the derivative of mass change.

Theoretical CO_2 capturing capacity. The theoretical capacity of the forced carbonation process (to offset CO_2 production during the preparation of OPC) was determined based on a stoichiometric analysis of the hydration reaction in the presence of abundant carbon dioxide to mimic the effects of forced out-of-equilibrium carbonation. Traditionally, OPC is a mixture of limestone and clay that is calcined at high temperatures (1450°C) and ground to produce a multiphase clinker (primarily alite and belite) (Eq. 1):

$$3CaCO_3 + SiO_2 \xrightarrow{1450°C} (CaO)_3SiO_2 + 3CO_2 \uparrow \quad (1)$$

Because of the intrinsic alkaline nature of the products formed from clinker hydration (Eq. 2 for Ca/Si ratio of C-S-H equal to 1.7), the hardened cement serves as a significant CO_2 absorbing agent through natural carbonation of calcium hydroxide (CH):

$$(CaO)_3SiO_2 + 2.3H_2O \rightarrow (CaO)_{1.7} \cdot SiO_2 \cdot H_2O + 1.3Ca(OH)_2 \quad (2)$$

The hydration of alite and belite in presence of carbonate ions leads to the formation of both calcium carbonate ($CaCO_3$) and a C-S-H gel. E.g., for alite we have:

$$3CaO \cdot SiO_2 + (3\text{-}x)CO_2 + y\,H_2O \rightarrow x\,CaO \cdot SiO_2 \cdot y\,H_2O + (3\text{-}x)CaCO_3 \quad (3)$$

Furthermore, any portlandite formed will convert over time into $CaCO_3$ if carbon dioxide is dissolved in excess:

$$Ca(OH)_2 + CO_2 \rightarrow CaCO_3 + H_2O \quad (4)$$

Dataset S1 (separate file). A Matlab script (Correlation_functions.m) was created to evaluate the correlation functions (two-point auto-correlation ($S_2(r)$) and cross-correlation ($X_2(r)$) functions) to quantitatively represent the phase distributions and their spatial correlation at progressing stages of hydration.

SI References

1. W. C. Oliver, G. M. Pharr, An improved technique for determining hardness and elastic modulus using load and displacement sensing indentation experiments. *J. Mater. Res.* **7**, 1564–1583 (1992).
2. H.-C. Loh, H.-J. Kim, F.-J. Ulm, A. Masic, Time-Space-Resolved Chemical Deconvolution of Cementitious Colloidal Systems Using Raman Spectroscopy. *Langmuir* **37**, 7019–7031 (2021).
3. M. Thiery, G. Villain, P. Dangla, G. Platret, Investigation of the carbonation front shape on cementitious materials: Effects of the chemical kinetics. *Cem. Concr. Res.* **37**, 1047–1058 (2007).
